# The Application of Fluorescence Anisotropy for Viscosity Measurements of Small Volume Biological Analytes

**DOI:** 10.3390/jeta1020007

**Published:** 2023-12-01

**Authors:** Matthew J. Sydor, Monica A. Serban

**Affiliations:** 1 BioSpectroscopy Core, Center for Biomolecular Structure and Dynamics, University of Montana, Missoula, MT 59812, USA; 2 Department of Biomedical and Pharmaceutical Sciences, University of Montana, Missoula, MT 59812, USA; 3 Montana Biotechnology Center (BIOTECH), University of Montana, Missoula, MT 59812, USA

**Keywords:** fluorescence anisotropy, viscosity, biological fluids

## Abstract

Time-resolved fluorescence anisotropy has been extensively used to detect changes in bimolecular rotation associated with viscosity levels within cells and other solutions. Physiological alterations of the viscosity of biological fluids have been associated with numerous pathological causes. This current work serves as proof of concept for a method to measure viscosity changes in small analyte volumes representative of biological fluids. The fluorophores used in this study were fluorescein disodium salt and Enhanced Green Fluorescent Protein (EGFP). To assess the ability of the method to accurately detect viscosity values in small volume samples, we conducted measurements with 12 μL and 100 μL samples. No statistically significant changes in determined viscosities were recorded as a function of sample volume for either fluorescent probe. The anisotropy of both fluorescence probes was measured in low viscosity standards ranging from 1.02 to 1.31 cP, representative of physiological fluid values, and showed increasing rotational correlation times in response to increasing viscosity. We also showed that smaller fluid volumes can be diluted to accommodate available cuvette volume requirements without a loss in the accuracy of detecting discrete viscosity variations. Moreover, the ability of this technique to detect subtle viscosity changes in complex fluids similar to physiological ones was assessed by using fetal bovine serum (FBS) containing samples. The presence of FBS in the analytes did not alter the viscosity specific rotational correlation time of EGFP, indicating that this probe does not interact with the tested analyte components and is able to accurately reflect sample viscosity. We also showed that freeze–thaw cycles, reflective of the temperature-dependent processes that biological samples of interest could undergo from the time of collection to analyses, did not impact the viscosity measurements’ accuracy. Overall, our data highlight the feasibility of using time-resolved fluorescence anisotropy for precise viscosity measurements in biological samples. This finding is relevant as it could potentially expand the use of this technique for in vitro diagnostic systems.

## 1. Introduction

Viscosity changes to biological fluids such as blood, saliva, lung surfactant, or synovial fluid have been associated with a variety of diseases and conditions [1–4]. Plasma and cerebrospinal fluid viscosity has been extensively employed as a fluid biomarker for various pathologies [5]. In particular, increases in blood viscosity associated with blood hyperviscosity syndrome, Sickle cell disease, polycythemia, hyperproteinemia, or hyperlipidemia result from an abnormally high number of red or white blood cells, or an increase in serum proteins or lipids [6–10]. Blood viscosity is strongly correlated with blood flow, with an increase in blood viscosity translating to three times the inverse change in blood flow. Blood viscosity has also been associated with other diseases such as myocardial infarction, venous thrombosis, and malaria [2]. Accurate assessments of blood viscosity are therefore important in understanding associated risk factors and potential correlated pathologies [11].

Viscosity changes in saliva have similarly been associated with disease. Burning mouth syndrome is a chronic painful condition that has been associated with lower salivary viscosity, especially in women [1]. This condition is detrimental to the mental and physical health of the patients by resulting in decreased oral function and the development of depression and anxiety [1]. Conversely, Sjogren’s syndrome, an autoimmune disease that affects the salivary and lacrimal glands, is associated with increased salivary viscosity. Diabetes and cystic fibrosis are also characterized by abnormal salivary viscosities [12–14]. Therefore, salivary viscosity could be considered a marker of several abnormal conditions and a characteristic of salivary function.

Lung surfactant is another biofluid with a direct correlation between viscosity and function which has been associated with disease [15–17]. The purpose of lung surfactant is to facilitate breathing by lowering the surface tension at the air–liquid interface in the lungs [18]. Alterations to the viscosity of this fluid impact the breathing ability of individuals and are associated with pathologies such as cystic fibrosis or chronic obstructive pulmonary disease [14,19]. Studies to develop artificial lung surfactants to treat preterm infants with difficulty breathing are also being conducted, in an approach described as surfactant replacement therapy [20]. Thus, considering that the viscosity of respiratory fluids is essential for the maintenance of normal physiological effects, it could serve as a potential pathological biomarker.

Lastly, viscosity changes in synovial fluid—a thick solution that lubricates joints—have been associated with arthritis. This inflammatory condition causes joint stiffness and pain [21,22] and viscosupplementation via joint injection of high-molecular-weight natural polymers is one of the treatment approaches [23,24]. While the collection of synovial fluid for diagnosis purposes is not feasible in the context of existing, less invasive diagnostic tools, this biological fluid represents yet another instance where viscosity changes are associated with disease.

While the correlation between the viscosity of the above biofluids and various diseases is now understood, there might be aberrant conditions, such as vestibular or cochlear disfunctions, where variations in perilymph or endolymph viscosity could be important but have never been interrogated due to both difficulties in obtaining such samples as well as sample volume limitations. For instance, imaging studies indicate that in humans the endolymph volume is 34 μL and the perilymph volume is 158 μL [25], with the volume of inner ear fluids in corresponding preclinical models substantially lower [25–27]. Therefore, the availability of techniques for accurately measuring the viscosity of small volumes of biofluids is of considerable importance.

Time-resolved fluorescence anisotropy is a technique that determines the rotation of a fluorophore by measuring polarized emissions. It has been used extensively to assess the rotational correlation times of biomolecules, which are sensitive to the viscosities of the solvent or medium [28,29]. By using fluorescent molecules with known rotational correlation times, insights into viscosity changes in solutions can be gained. Two commonly used fluorophores of different molecular weights are Enhanced Green Fluorescent Protein (EGFP) [32.7 kDa] and fluorescein [376 Da]. The effect of viscosity on the rotational correlation time of GFP has been demonstrated with aqueous solutions containing viscogens such as glycerol and polyethylene glycol [30,31]. For example, in buffers with a viscosity of 1.0 cP, GFP has rotational correlation times in the range of 14–16 ns, which shifts to >50 ns with the addition of glucose and fructose or glycerol [30,31]. A GFP analog, Enhanced GFP (EGFP), has also been previously employed as a probe in techniques such as fluorescence recovery after photobleaching (FRAP), fluorescence anisotropy, and fluorescence correlation spectroscopy (FCS) in mammalian and bacterial cytosol samples, in viruses, and aqueous solutions [32–34]. Also, the effects of pH on the excited state dynamics of EGFP have been determined and, as such, the probe has been utilized for sensing pH changes [35]. Separately, fluorescein has been employed as a pH sensor and viscometer. The spectral properties of fluorescein have been observed to change with the addition of viscogens such as sucrose and glycerol, as well as with alterations to pH [36], and this molecule is also routinely used in diagnostic tests such as retinal angiography [37]. A fluorescein derivative, fluorescein-isothiocyanate (FITC), is a commonly used label of biomolecules that allows for studying viscosity and the diffusion of these molecules [38,39]. For example, one study measured viscosity in vitreous humor biofluids by using FITC-labelled dextrans, ficolls, and bovine serum albumin (BSA) [38]. Another study used FITC dextrans to measure diffusivity in contracted collagen gels [39].

In the current study, we demonstrated the feasibility of employing time-resolved fluorescence anisotropy for detecting viscosity variations in biological fluids. Though no new biophysical discoveries are reported here, the study highlights the feasibility of using fluorescence anisotropy to probe small sample volumes and to detect subtle (less than 1 cP) differences in viscosity. As indicated, this technique could offer a valuable analytical tool for fields such as hearing research or in vitro diagnostics development.

We also assessed the relevance of this approach to analyze biofluid viscosity in small volumes (≤100 μL). As indicated previously, the available volume of biological samples can be of critical importance in determining what measurements can be performed on the samples. Here, fluorescein and EGFP were used as probes to measure viscosity changes in a series of physiologically relevant low viscosity standards. Samples were measured in 12 μL volume cuvettes and data were compared to samples measured in 100 μL cuvettes in order to demonstrate that the viscosity values obtained were volume independent. It is important to note, however, that this volume range was imposed by the size/volume of cuvettes commercially available to us and does not actually reflect the methodology’s limitation. However, we showed that smaller fluid volumes (4 μL) can be diluted to accommodate cuvette volume requirements (12 μL) without a loss in the ability of the technique to adequately detect viscosity differences between samples. As a biological fluid model, we tested samples containing fetal bovine serum (FBS). We used this model to represent samples that could potentially benefit from this technique. Additionally, we evaluated the effects of multiple freeze–thaw cycles on sample viscosity measurements to demonstrate that anisotropy/sample viscosity results were not affected by the temperature variations that biofluid samples could undergo during transportation and/or storage. Overall, this study describes a method that allows accurate biofluid viscosity detection in limited sample volumes (≤100 μL) and highlights its potential for yet-unexplored biofluid biomarker-based diagnostics.

## 2. Materials and Methods

### 2.1. Preparation of Viscosity Standards

The fluorescent dyes used in this study were fluorescein disodium salt from Alfa Aesar (Ward Hill, MA, USA) and recombinant EGFP from Novus Biological (NBP1–99915, Centennial, CO, USA) with N- and C-terminal His tags. The fluorescein was dissolved in Milli-Q ultrapure water to a concentration of 50 μM, while EGFP was reconstituted with Dulbecco’s phosphate-buffered saline from VWR (DPBS, VWRL0119–1000, Radnor, PA, USA) to a stock concentration of 30 μM. Each of the respective dyes were diluted into a set of low viscosity standards (low viscosity range kit, Ursa Bioscience, Bel Air, MD, USA. Fluorescein was used at a final concentration of 1 μM, while EGFP was used at a final concentration of 600 nM. When not measuring viscosity in the set of standards, DPBS was used to dilute the probes. The fluorescence cuvettes used were Hellma microor ultra-micro cuvettes (Hellma GmbH, Mullheim, Germany) with chamber volumes of 100 μL or 12 μL, respectively.

### 2.2. Time-Resolved Fluorescence Anisotropy Measurements

Changes to the rotational correlation times of fluorescein and EGFP were used as a measure of sample viscosity. Rotational correlation times were acquired from time-resolved fluorescence anisotropy measurements. These measurements were taken on a custom-built fluorimeter with parts from Quantum Northwest (Liberty Lake, WA, USA) and Edinburgh Instruments (Livingston, UK). A PicoQuant 470 nm pulsed diode laser (5 MHz repetition rate, LDH-P-C-470, PicoQuant GmbH, Berlin, Germany) was used to excite either the fluorescein or EGFP. Data were acquired at 3000 counts per second (cps) for a duration of 5 min in order to obtain a maximum of at least 10,000 counts for vertically polarized emissions. This integration time was kept the same for horizontal emissions. Horizontal excitation with vertical emissions (HV) and horizontal excitation with horizontal emissions (HH) were used to measure G-factor, which was determined by integrating HV and HH. The instrument response functions (IRFs) were generated with the 470 nm laser and scattering solution with no emission filter present. The IRF was allowed to integrate until 50,000 max counts were reached. The emissions were then gated with a 500 lp filter from Chroma (Bellows Falls, VT, USA) and detected with an Edinburgh Instruments PMT (Livingston, UK). All data were collected at a temperature of 20 °C +/− 0.01. A PicoQuant MultiHarp 150 with 80 picoseconds (ps) resolution (Berlin, Germany) was used as the TCSPC module and anisotropy data were fit with an exponential reconvolution model using PicoQuant FluoFit software (version 4.6.6, PicoQuant GmbH, Berlin, Germany). Examples of the fitted data are shown in Supplementary Figures S1 and S2. The time-resolved fluorescence anisotropy is given by Equation (1), where r(t) is the anisotropy and $I_{VV}$ and $I_{VH}$ are the intensities from vertical and horizontally polarized emissions, respectively, and G is the G-factor, which was determined from integrating HH and HV.


(1) $$r(t)=\left[I_{VV}(t)-GI_{VH}(t)\right]/\left[I_{VV}(t)+2GI_{VH}(t)\right]$$

The rotational correlation time of the molecule can be calculated by Equation (2). In this equation, $r_{0}$ is the initial anisotropy at time zero and θ is the rotational correlation time. This equation is based on the rotational properties of a sphere and is relevant for fluorophores that behave as such. Herein, EGFP and fluorescein behave in a manner similar to that of a sphere. It is also important to consider the fluorescence lifetime of the probe in relation to the molecular weight. A rotational correlation time much more than 10 times the fluorescence lifetime can be difficult to measure as the t/θ ratio becomes close to zero, causing r to approach $r_{0}$. The rotational correlation time of EGFP in relation to the fluorescence lifetime is only about 7–10 times larger; therefore, accurate rotational correlation times in relation to the literature can still be obtained. On the other hand, as θ becomes much smaller than t,r then approaches zero. In such cases, fast dynamics can be difficult to accurately measure depending on the resolution of the event timing system.


(2) $$r(t)=r_{0}e^{-t/\theta }$$

Equation (3) shows the relationship between viscosity and the rotational correlation time. Here, η is the viscosity, V is the volume of the molecule, k is the Boltzmann constant, and T is the temperature.


(3) θ=ηV/kT

## 3. Results and Discussion

### 3.1. Viscosity Measurements in Different Analyte Volumes

In order to assess whether fluorescence anisotropy measurements could be applied to small volumes of biological samples, initial measurements were taken with two different sample volumes, 12 μL and 100 μL. Fluorescein or EGFP, employed herein as viscosity probes, were diluted in a series of low viscosity standards to demonstrate their ability to reflect small viscosity changes.

**Figure 1.**
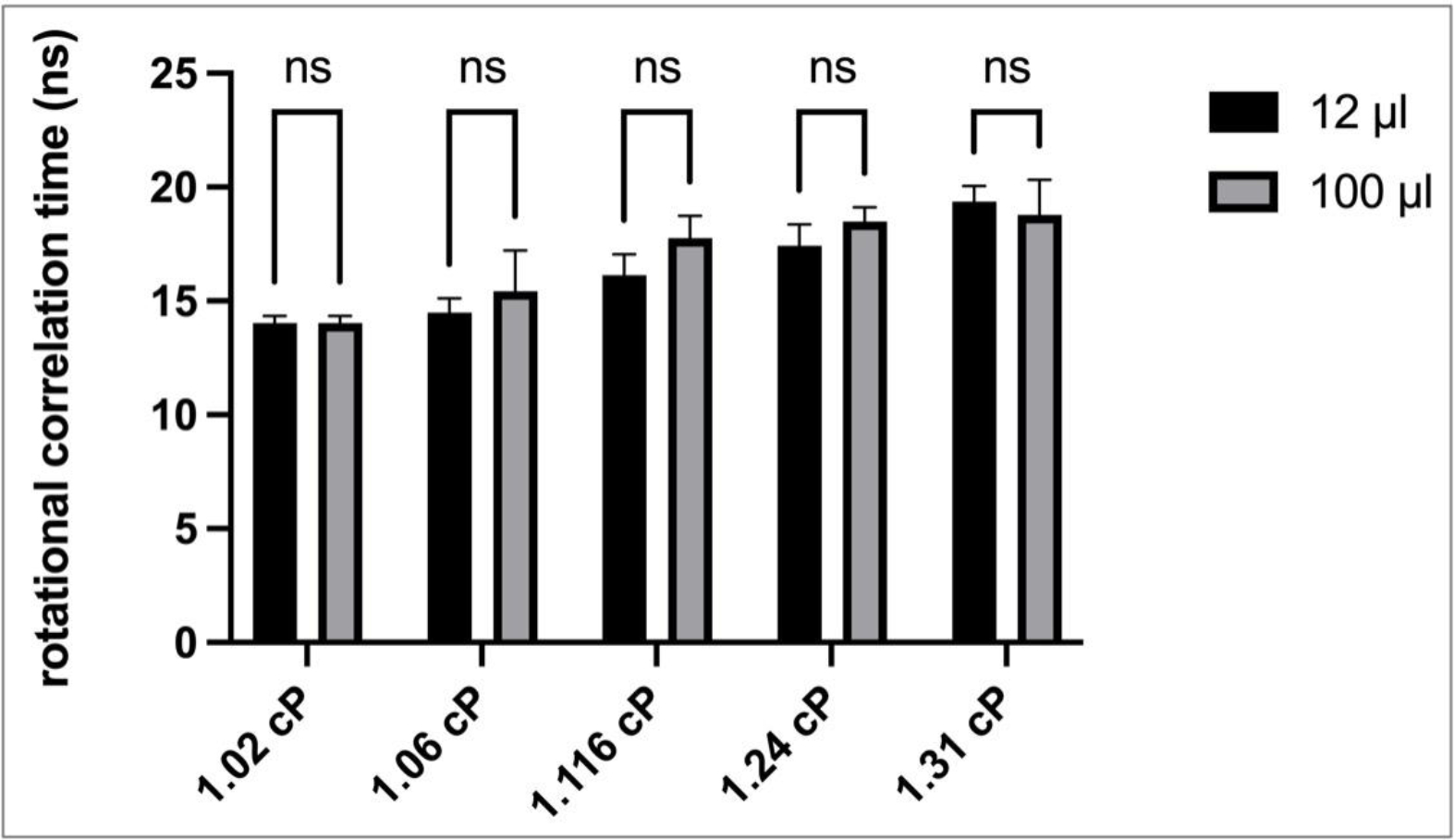
Time-resolved anisotropy measurements of EGFP in different sample volume sizes. Rotational correlation times were derived from time-resolved fluorescence anisotropy measurements of EGFP at different viscosities. The measurements collected with two different analyte volumes (12 μL and 100 μL); ns—not significant; n = 3–4; error bars illustrate standard deviation (SD).

For EGFP, the rotational correlation times calculated from time-resolved anisotropy measurements in both sample volumes are displayed in Figure 1.

The small volume (12 μL) EGFP data showed an increasing rotational correlation time with increasing viscosity, which correlated with a similar pattern observed in larger analyte volume (100 μL) measurements. A two-way ANOVA with Sidak post-test was used to compare the viscosity means of these data. Our analysis revealed no significant differences, indicating that analyte volume does not affect the accuracy anisotropy measurements. These results indicate that discrete changes in analyte viscosity as exemplified by the use of low viscosity standards, regardless of the sample volume used for the analyses, could be clearly and accurately detected with this methodology. Moreover, the data highlight the feasibility of using fluorescence anisotropy as a tool for the evaluation of small volume biological samples, without the need for extensive manipulations.

### 3.2. Viscosity Measurements in Different Analyte Volumes with Alternate Probe

**Figure 2.**
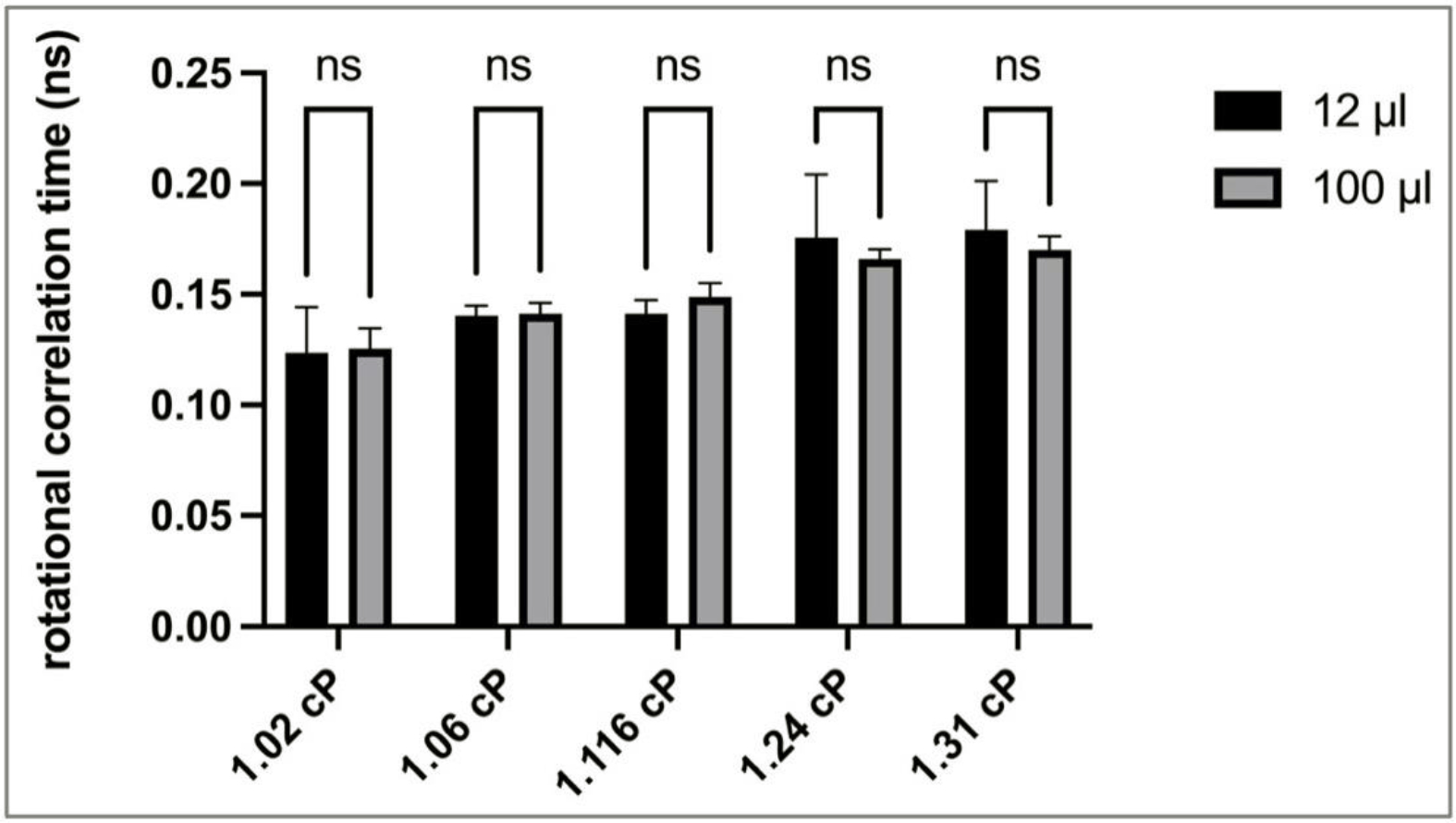
Analysis of the effect of sample volumes on fluorescein anisotropy measurements. A two-way ANOVA with Sidak post-test was used to compare the mean rotational correlation time of each cuvette size in each viscosity standard. ns—not significant; n = 3–4; error bars illustrate SD.

As an alternative viscosity probe, we also assessed fluorescein because of its smaller molecular weight and different chemical nature compared to EGFP. Figure 2 illustrates the viscosity measurement data as detected via fluorescein anisotropy with fluorescein as the probe in small and larger analyte volumes. As expected, the rotational correlation times of fluorescein increased as the viscosity of the standards increased. A side-by-side comparison of the rotational correlation time values detected via fluorescein anisotropy in 12 μL and 100 μL standard samples revealed no analyte volume-specific significant differences in viscosity values, similar to our findings with the EGFP probe. Cumulatively, our analyses with the two different probes further underline the independence of the measured viscosity values from the analyte volumes. Based on these findings, for convenience, further analyses presented herein were conducted with 100 μL sample volumes.

The rotational correlation times of both EGFP and fluorescein produced a linear trend when plotted against viscosity. These are shown as Supplementary Figures S3 and S4.

### 3.3. Viscosity Measurements in Diluted Samples

**Figure 3.**
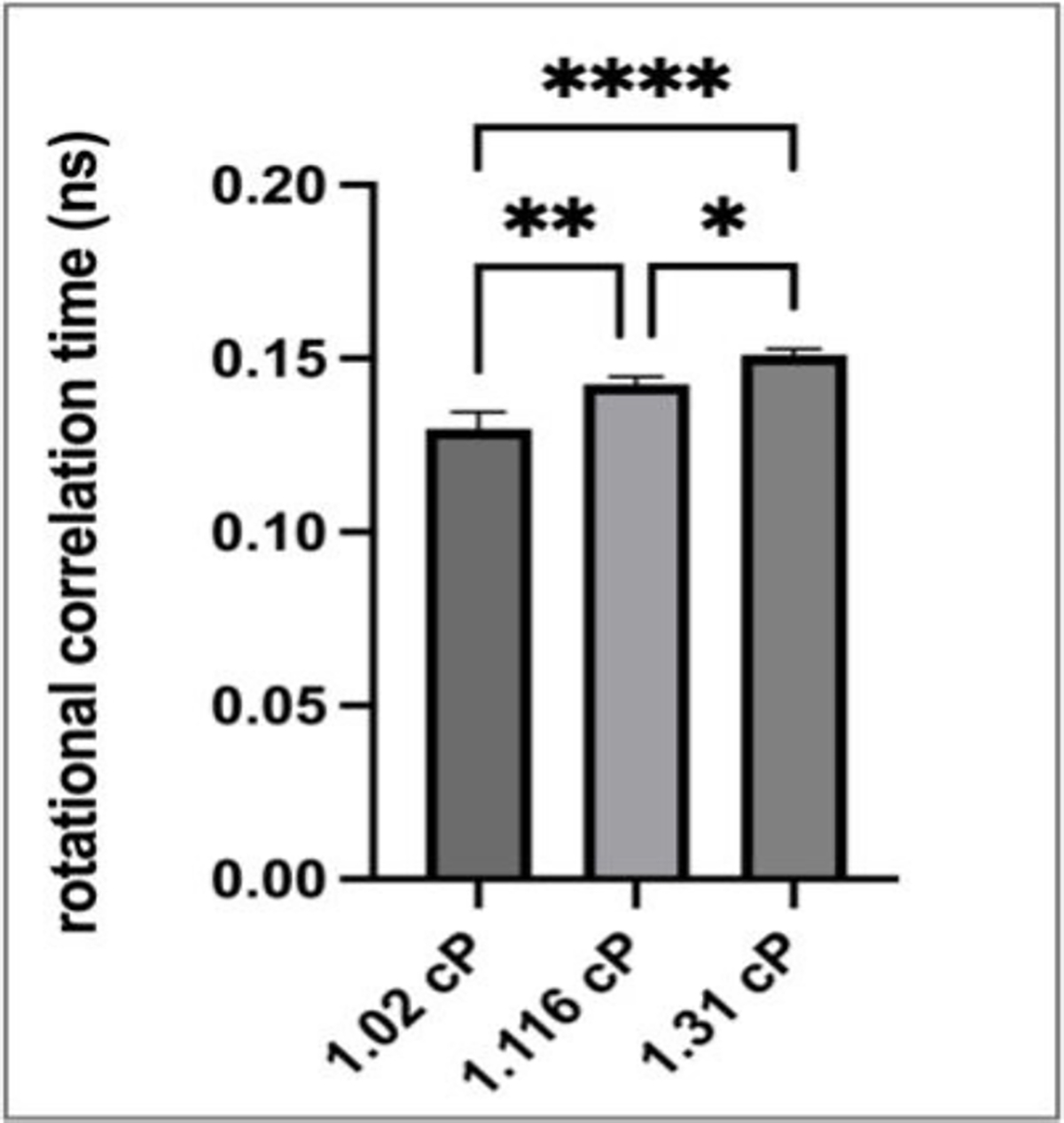
Viscosity measurements in fluorescein-diluted samples. Viscosity standards of 1.02, 1.116, 1.31 cP, 4 μL of each, were diluted with DBPS to a volume of 12 μL. n = 3–4; a one-way ANOVA with Tukey multiple comparison test was used to determine significance * *p* < 0.5, ** *p* < 0.01, **** *p* < 0.0001; error bars illustrate SD.

We next sought to understand if smaller fluid volumes can be diluted to accommodate cuvette volume requirements of 12 μL without a loss in the ability of the technique to adequately detect small viscosity differences between viscosity standards in the 1.02–1.31 cP range. For this, 4 μL of 1.02, 1.116, and 1.31 cP viscosity standards were mixed with 8 μL of DPBS corresponding to a 3X dilution, and then rotational correlation times using fluorescein as a probe were determined. The data presented in Figure 3 show that discrete differences in sample viscosities can still be effectively determined. Moreover, for actual biological sample analyses, low viscosity standards could be used to build standard curves, and the determined viscosity values in diluted samples could be extrapolated to their actual non-diluted values.

### 3.4. Rotational Correlation Times Analyses

**Figure 4.**
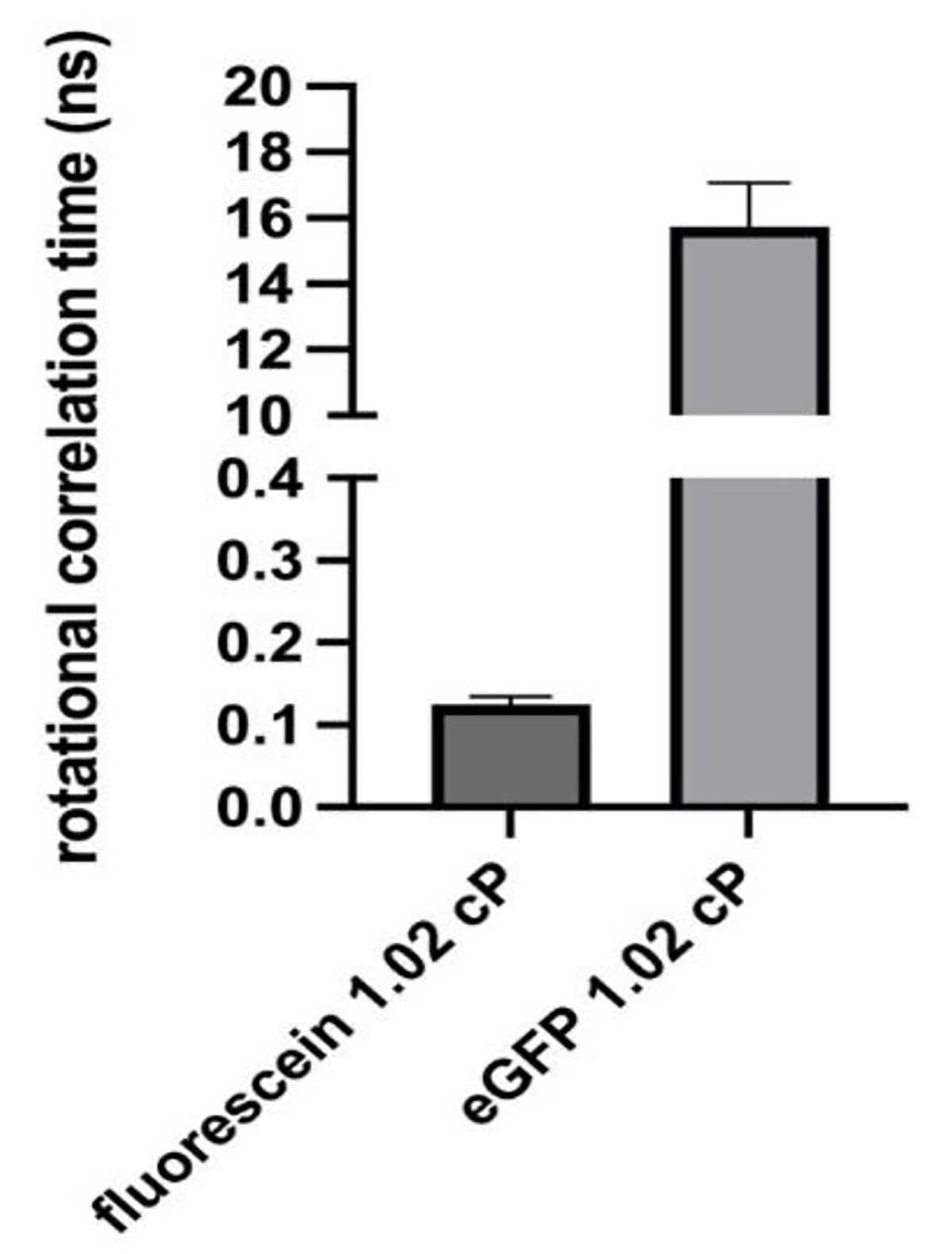
Time-resolved anisotropy measurements of fluorescein end EGFP in 100 μL samples 1.02 cP viscosity standard. Rotational correlation times are specific to the experimental probe used; n = 3–4; error bars illustrate SD.

Figure 4 displays the correlation times of fluorescein and EGFP in the 1.02 cP low viscosity standard. The rotational correlation time for various forms of GFP has been previously reported to be from approximately 16 ns to greater than 20 ns, depending on the experimental conditions [30–32,40]. In the present study, the EGFP rotational correlation times ranged from averages of 15.7 ns in the 1.02 cP standard to 19.37 ns in the 1.31 cP standard; these values are consistent with the range reported in the literature, confirming the accuracy of our measurements. The much lower values which correlate with the use of fluorescein, which are expected based on its molecular weight, also correlate with previously published data (picosecond timescale) [41], additionally confirming the validity of our measurements. While both probes used in this study appear to work well for viscosity assessments in small volume samples, it is important to note that fluorescein’s fluorescent properties are pH dependent, which may pose challenges for certain biological analytes [42]. Therefore, for our subsequent experiments, we focused on using EGFP as a probe.

### 3.5. Relevance of Technique to Biological Fluid Viscosity Measurements

**Figure 5.**
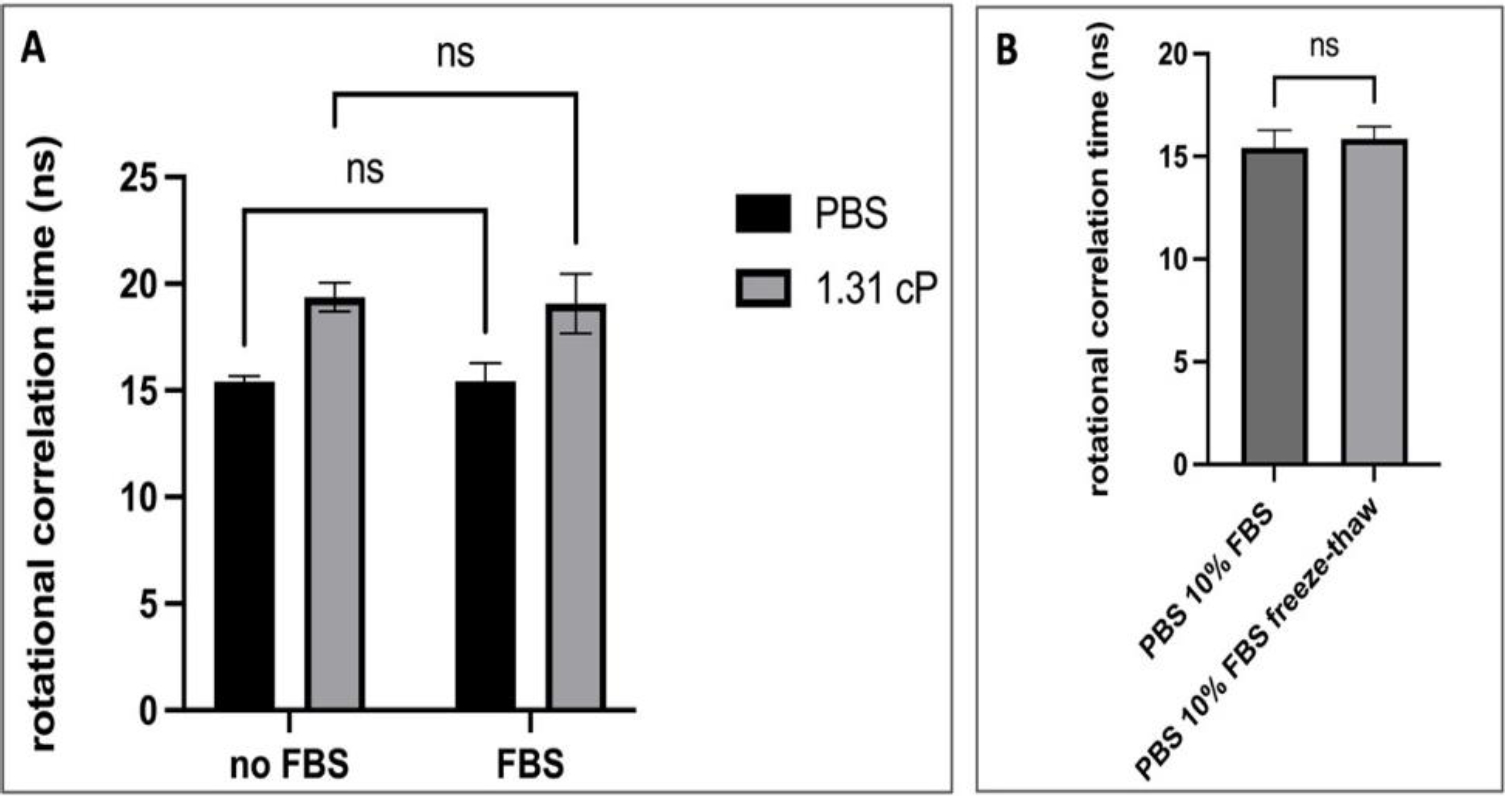
The response of EGFP rotational correlation times in a biological sample mimetic. (**A**)—comparison of rotational correlation times in samples with or without FBS; (**B**)—evaluation of freeze–thaw effects rotational correlation times in samples with FBS. A two-way ANOVA with Sidak post-test was used to compare the data. ns—not significant; n = 3–4; error bars illustrate SD.

To further assess the utility of fluorescence anisotropy measurements in small volume biological samples, EGFP was added to either DPBS with 10% *v/v* FBS or to the 1.31 cP viscosity standard with 10% FBS (Figure 5A). Separately, a solution of DPBS with 10% FBS was refrozen and then thawed after every experimental replicate to simulate the temperature-dependent processes that a potential analyte could undergo from the time of the sample collection to sample analysis (Figure 5B). Our data indicate that the addition of FBS to a final concentration of 10% did not alter the rotational correlation time of EGFP in either DPBS or the 1.31 cP viscosity standard (Figure 5A). This result indicates that EGFP is capable of detecting subtle (less than 1 cP) viscosity changes, even in biological fluids with a complex composition such as FBS. Additionally, multiple freeze–thaw cycles did not appear to impact the rotation of the EGFP (Figure 5B). Promisingly, we were able to detect an increase in the rotational correlation times between the 1.02 cP PBS sample and the 1.31 cP sample (both with or without FBS), which mirrors the results presented in Figure 1 and confirms that discrete viscosity changes are detectable in complex biological specimen representative samples. Fluorescein was not employed in the samples containing FBS in order to avoid interactions between the probe and serum components, which could confound the anisotropy results.

## 4. Conclusions

Overall, this study demonstrates the utility of two common and easily available fluorophores for viscosity detection applications in small volume biological samples via fluorescence anisotropy measurements. We have shown that analyses conducted in 100 μL samples versus almost 10-times smaller volumes (12 μL) yielded statistically identical results, highlighting the high sensitivity of the analysis technique and its independence from the analyte volume. Moreover, we found that smaller fluid volumes can be diluted to accommodate cuvette volume requirements, with statistically significant viscosity differences between samples still effectively detectable. The smallest sample volume employed in this study was dictated by the commercial availability of analysis cuvettes. The availability/development of even smaller size analyte receptacles could potentially further decrease the amount of analyte volume needed for analysis without any anticipated loss of measurement accuracy.

Additionally, the investigation of the two different probes, one a small dye with a molecular weight of 376 Da and the other a protein of 32.7 kDa, allowed us to probe the extent of their interaction with the analyte components and offered a glimpse into considerations that would be pertinent for various types of biological sample analyses. As mentioned, the use of fluorescein might have limitations given the pH dependence of its fluorescent properties; also, cost efficiency and ease of procurement might be additional factors that inform probe selection. Nonetheless, our cumulative data favorably position this technique to be further considered for miniaturization and portability development efforts, especially for small volume biological sample analyses.

## Supplementary Material

jeta-2664281-supplementary 2

## Data Availability

The data presented in this study are available on request from the corresponding author.
