# Supplementary material for: The Application of Fluorescence Anisotropy for Viscosity Measurements of Small Volume Biological Analytes
Source: J Exp Theor Anal. Author manuscript; Available in PMC 2024 Apr 17. (PMC11022525; doi:10.3390/jeta1020007)
Supplement: jeta-2664281-supplementary 2 [file NIHMS1981275-supplement-jeta-2664281-supplementary_2.pdf]

Supplementary Materials: The Application of Fluorescence Anisotropy for Viscosity Measurements of Small Volume Biological Analytes

Matthew J. Sydor and Monica A. Serban

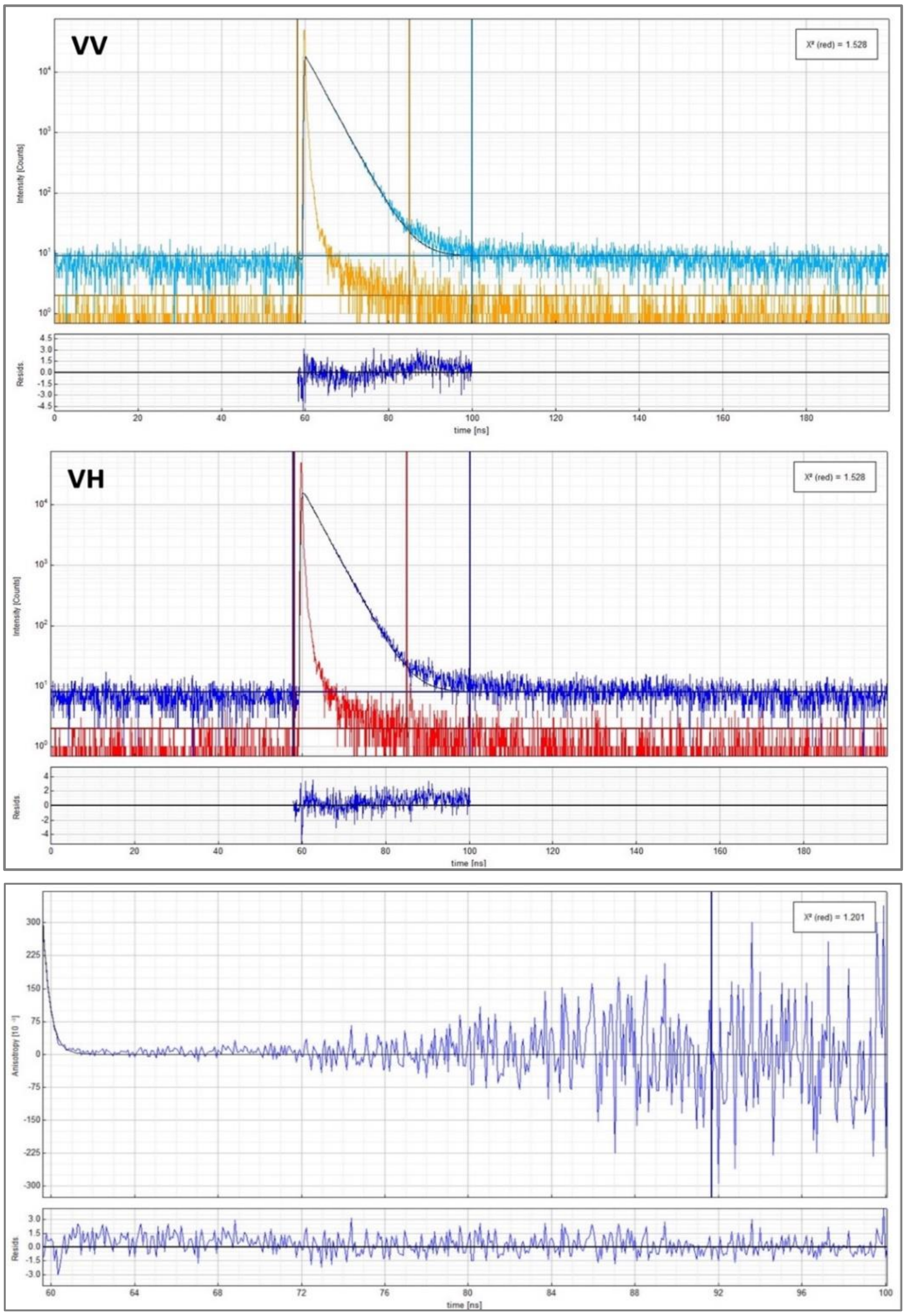

Figure S1. Example of fitted fluorescein anisotropy decays. Vertically polarized emissions (VV) are shown on top, while horizontal emissions (VH) are shown in the middle graph. An example anisotropy decay is shown in the bottom graph. The instrument response functions are plotted in yellow (VV) and red (VH). Each decay is fitted with an exponential reconvolution. The residuals displaying the quality of fit are shown at the bottom of each graph, with the resulting chi square value at the top right. A value closer to one represents a better fit of the data.

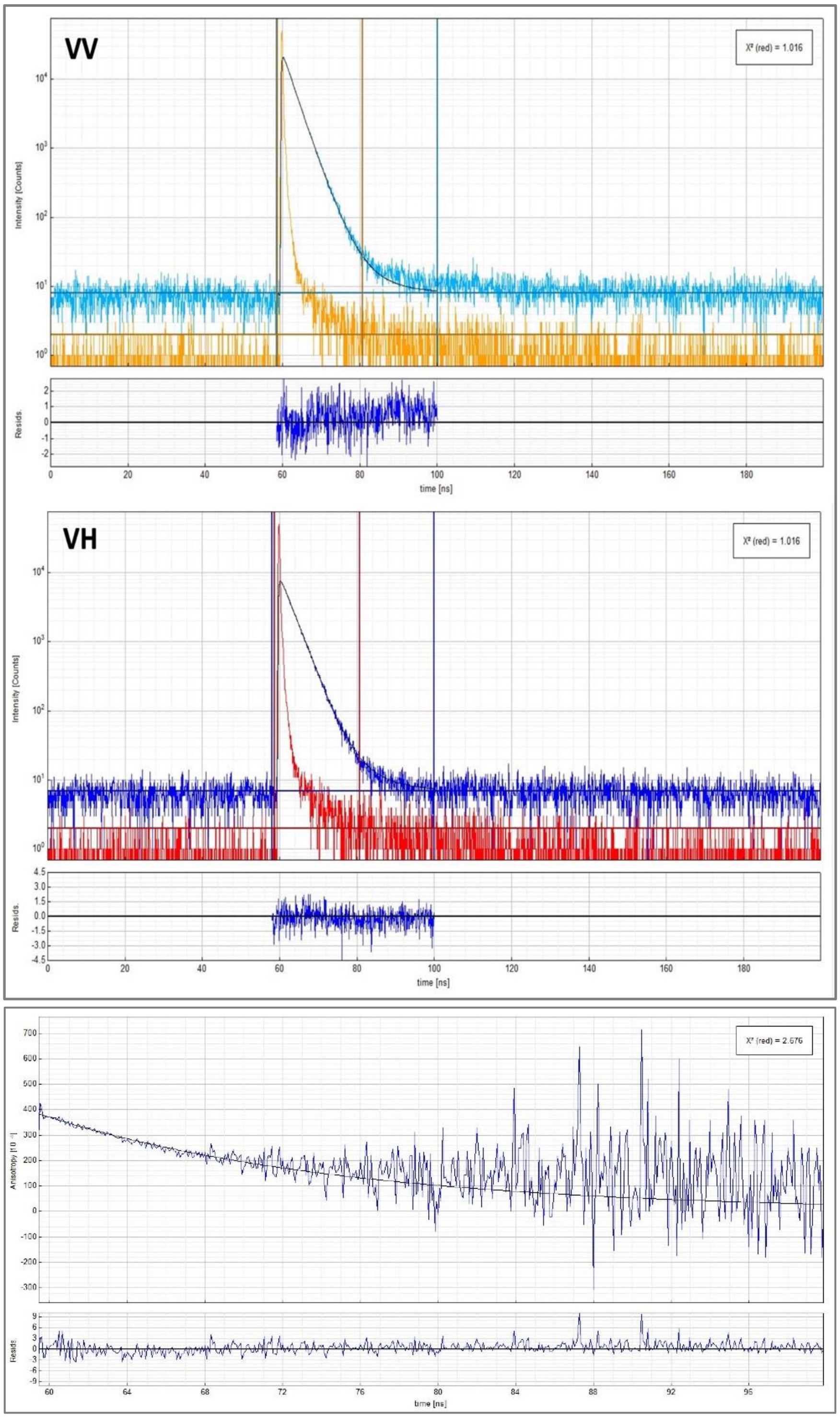

Figure S2: Example of fitted EGFP anisotropy decays. Vertically polarized emissions (VV) are shown on top, while horizontal emissions (VH) are shown in the bottom graph. An example anisotropy decay is shown in the bottom graph. The instrument response functions are plotted in yellow (VV) and red (VH). Each decay is fitted with an exponential reconvolution. The residuals displaying the quality of fit are shown at the bottom of each graph, with the resulting chi square value at the top right. A value closer to one represents a better fit of the data.

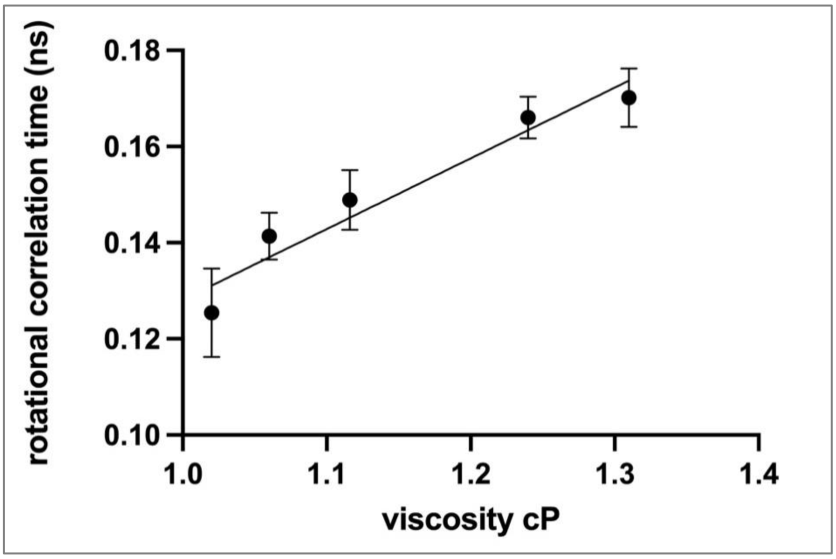

Figure S3: Fluorescein rotational correlation times plotted vs. viscosity. The rotational correlation times of fluorescein in each of the 1.02-1.31 cP viscosity standards were plotted with a linear regression fit to demonstrate the trend in rotational correlation times with increasing viscosity. Plotted points are mean +/− SEM. n = 3–4.

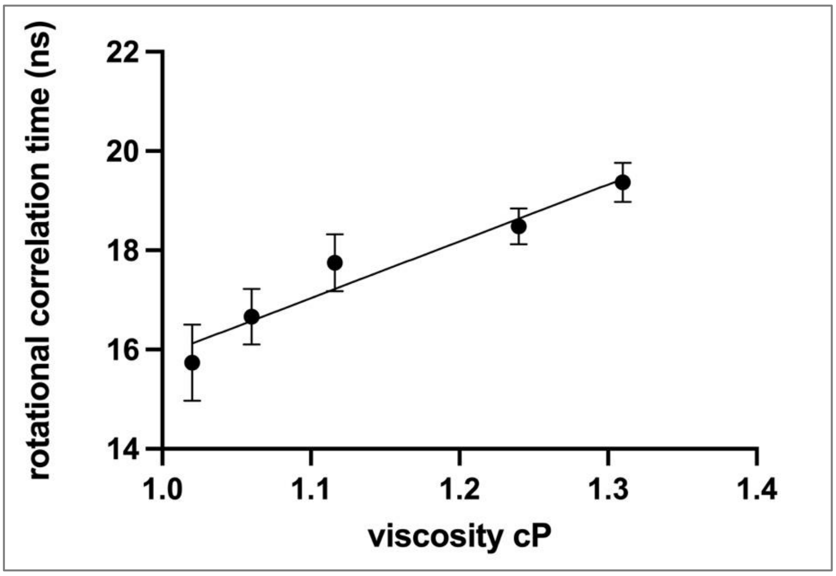

Figure S4: EGFP rotational correlation times plotted vs. viscosity. The rotational correlation times of EGFP in each of the 1.02-1.31 cP viscosity standards were plotted with a linear regression fit to demonstrate the trend in rotational correlation times with increasing viscosity. Plotted points are mean +/− SEM. n = 3–4.
